# The Q-rich/PST domain of the AHR regulates both ligand-induced nuclear transport and nucleocytoplasmic shuttling

**DOI:** 10.1038/srep32009

**Published:** 2016-08-18

**Authors:** Anna Tkachenko, Frank Henkler, Joep Brinkmann, Juliane Sowada, Doris Genkinger, Christian Kern, Tewes Tralau, Andreas Luch

**Affiliations:** 1 German Federal Institute for Risk Assessment (BfR), Department of Chemical and Product Safety, Max-Dohrn-Strasse 8-10, 10589 Berlin, Germany

## Abstract

The aryl hydrocarbon receptor (AHR) shuttles continuously between cytoplasm and nucleus, unless ligand-binding triggers association with the AHR nuclear translocator (ARNT) and subsequent binding to cognate DNA motifs. We have now identified Val 647 as mandatory residue for export from the nucleus and AHR-function. This residue prevents inactivation of the receptor as a consequence of nuclear sequestration *via* constitutive import. Concomitantly mutants lacking this residue are exclusively localised in the nucleus. Although ligands accelerate nuclear import transiently, stable nuclear transition depends on a motif adjacent to Val 647 that comprises residues 650–661. Together, this defined region within the Q-rich domain regulates intracellular trafficking of the AHR in context of both nucleocytoplasmic shuttling and receptor activation. Nuclear export therefore depends on the previously characterised N-terminal NES and the newly identified motif that includes V647. Nucleocytoplasmic distribution of full-length human AHR is further affected by a section of the PST domain that shows sequence similarities with nuclear export signals. In concert, these motifs maintain a predominant cytoplasmic compartmentalisation, receptive for ligand binding.

The AHR is a highly conserved protein belonging to the basic helix-loop helix (bHLH)-PAS family^1^,^2^. Originally identified for its association with xenobiotic ligands, particularly 2,3,7,8-tetrachlorodibenzo-*p*-dioxin (TCDD)^3^, this receptor became quickly recognised as one of the major regulators for eukaryotic phase-I metabolism. Further xenobiotic ligands include β-naphthoflavone (βNF), polychlorinated biphenyls, as well as carcinogenic polycyclic aromatic hydrocarbons (PAHs)^4^. Amongst other effects receptor activation induces expression of key enzymes of oxidative phase-I metabolism, notably cytochrome P450-dependent monooxygenases (CYPs) 1A1 and 1B1, both of which convert benzo[*a*]pyrene and other carcinogenic PAHs into highly mutagenic diol-epoxide intermediates^5^. Since then, certain endogenous ligands^6^,^7^ and additional properties of the AHR have been widely explored. These studies revealed important physiological functions in the immune response^8^, autoimmunity^9^,^10^, carcinogenesis^11^, apoptosis^12^, skin differentiation^13^, as well as in endocrine^14^,^15^ and host-microbiome^16^ signalling. Yet, comparatively little is known about its activation mechanisms that are tightly related with intracellular trafficking of receptor complexes. Notably, functional regulation might involve both nuclear import and export pathways. Prior to activation the AHR receptor is maintained in a cytoplasmic chaperone complex which consists of Hsp90, XAP2 and p23^17^. According to the current model, the ligand-bound AHR shifts to the nucleus and forms a heterodimer with ARNT to initiate transcription^18^. Importantly, constitutive nuclear translocation also occurs in the absence of ligand binding. This process is driven by ligand-independent import and balanced by parallel export in order to maintain a predominant cytoplasmic compartmentalisation. Although nucleocytoplasmic shuttling was recognised as early as 1998^19^, both the physiological relevance and molecular mechanisms are merely understood.

Activation and intracellular trafficking of the AHR are regulated by the N-terminal domain that contains a bipartite nuclear localisation signal (NLS)^19^, as well as an adjacent nuclear export signal (NES)^20^ and the DNA-binding domain^21^. The well-characterised NLS is both required and sufficient to mediate import of AHR receptor complexes, leading either to receptor activation or to re-export into the cytoplasm, especially in the absence of ligands. Importantly, amino acid residues 63–73 comprise an export signal (NES)^19^,^20^ which shows high sequence similarity to analogous viral and mammalian motifs^22^. This N-terminal NES overlaps with sequences that interact with the ARNT after ligand binding. It was hence postulated that nuclear export is blocked when this NES is masked by ARNT during receptor activation^23^. In consequence, AHR/ARNT complexes interact with xenobiotic response elements (XRE) to initiate expression of target genes^24^, including CYPs 1A1 and 1B1^25^. Further, nuclear export of the AHR was discussed to terminate transcription and shown to trigger degradation *via* cytoplasmic proteasomes^23^.

Several lines of evidence now suggest that nuclear export of the AHR depends on additional motifs or interacting factors. Notably, when the N-terminal NES was inactivated by mutagenesis, full-length AHR maintained a substantial cytoplasmic localisation in the absence of ligand^23^. In addition, several deletion mutants are exclusively localised in the nucleus^26^ although the NES was not affected. It was speculated that these variant patterns are related to distinct interactions with XAP2, protein modifications or alternate conformations^26^. Moreover, the human receptor shifts from cytoplasmic staining towards a substantial nuclear staining pattern, when the C-terminal domain is replaced by its murine homolog^27^. Still, data on if and how the C-terminal domain contributes to nucleocytoplasmic shuttling and AHR compartmentalisation remain scarce. In this study, we have addressed the role of nucleocytoplasmic shuttling in relation to AHR activation and demonstrate an essential regulation of both receptor activation and intracellular trafficking by the C-terminal domain.

## Results

### Nucleocytoplasmic shuttling occurs as a constitutive and dynamic process

**Figure 1:**
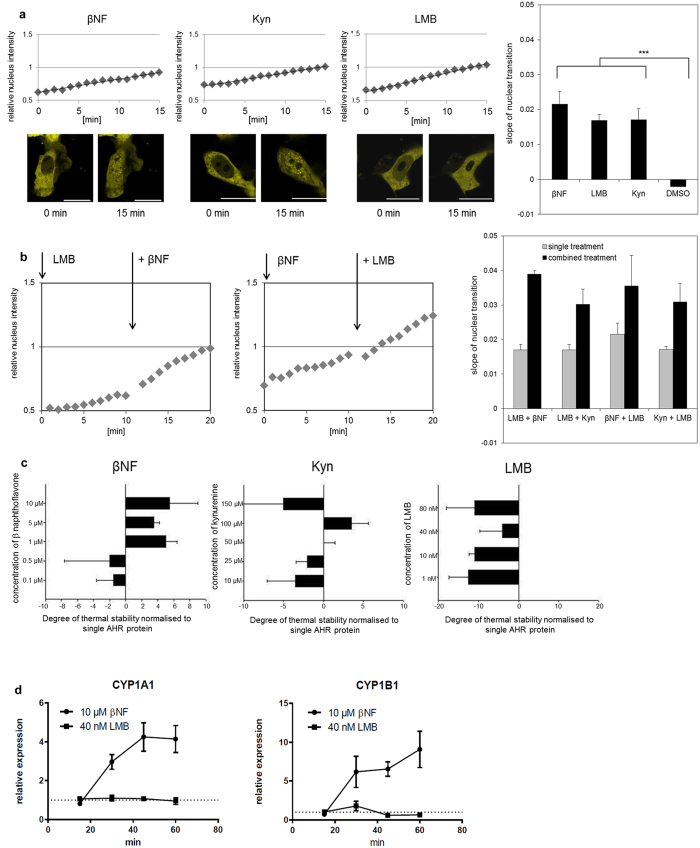
Constitutive nucleocytoplasmic shuttling of the AHR is accelerated by ligands. (**a**) Fluorescence images demonstrating the translocation of the full-length EYFP-AHR into the nucleus in HepG2 cells after treatment with 10 μM β-naphthoflavone (βNF), 100 μM kynurenine (Kyn) and 40 nM leptomycin B (LMB) for 15 min (scalebar = 20 μm). The selection of these concentrations was based on a dose-response analysis (Supplementary Fig. S2b). Graphs in the diagrams visualise the shift of nuclear staining in relation to total fluorescence of the analysed cells over 15 min. All three compounds triggered a comparable transition of EYFP-AHR into the nucleus, as indicated by comparable slopes of the graphs. Each bar (right diagram) represents the mean of at least 10 analysed cells +/− S.E.M. ***p < 0.001. (**b**) βNF accelerates the basal nucleocytoplasmic shuttling. Cells were treated for 10 min with 40 nM LMB, co-treated for another 10 min with 10 μM βNF (left side). Nuclear transition of EYFP-AHR was recorded and analysed as described above. Nucleocytoplasmic shuttling continues in the presence of ligand. Cells were treated with 10 μM βNF for 10 min, then co-treated with 40 nM LMB and analysed as described above (middle). Slopes of the recorded graphs were separately determined for single and combined treatments as indicated (right side). Each bar represents the mean of at least 10 cells +/− S.E.M. (**c**) Thermal shift assay with purified recombinant human AHR (protein purification is summarised in Supplementary Fig. S4). Shown is the relative thermal stability of AHR in the presence of LMB, Kyn and βNF at different concentrations. Compared to unliganded AHR, βNF and Kyn increased thermal protein stability, while LMB apparently has a destabilising effect. A decrease of stability was noted using 150 μM Kyn, possibly triggered by precipitation. Shown are the means of three biological replicates +/− S.E.M. (**d**) Time-dependent transcriptional activation of CYP1A1 and CYP1B1 in HepG2 cells. Induction was only seen after treatment with 10 μM βNF but not with 40 nM LMB. Displayed values represent relative inductions of transcripts normalised to the solvent control. Values shown are means of three biological replicates +/− S.E.M.

Previous investigations on the localisation of the AHR have mainly analysed cells that were fixed at defined time points and hence could only record cellular snap-shots. We therefore chose to investigate the cellular compartmentalisation of AHR in living HepG2 cells by live-cell fluorescence imaging using an expression construct that expresses the human matured protein (amino acids 11–848) fused to the C-terminus of enhanced yellow fluorescent protein (EYFP). The EYFP-tagged protein shows a shuttling behaviour similar to the wild-type protein, thus allowing continuous recording of functional AHR *in situ* (Fig. 1 and Supplementary Fig. S1). Our data confirm a constitutive and highly dynamic nucleocytoplasmic shuttling, since both, ligand binding of the xenobiotic βNF or endogenous kynurenine^6^ (Kyn), as well as export inhibition by leptomycin B (LMB) resulted in a comparable accumulation of AHR in the nucleus (Fig. 1a,b). The latter compound was isolated from fungi and is known to affect CRM1-mediated nuclear export^19^. The binding of the ligands βNF and Kyn was confirmed by measuring AHR stabilisation in a thermal-protein shift assay, as was the absence of binding for LMB (Fig. 1c). The latter also failed to induce AHR-dependent target genes (Fig. 1d), further confirming its solely inhibitory function on nuclear export. The predominant cytoplasmic localisation of AHR is therefore maintained as a steady state by means of continuous parallel import and export, but shifts to nuclear accumulation after application of ligands. In addition the kinetic data show that while ligand binding accelerates the basal constitutive import, partial export continues to occur (Fig. 1b). As a consequence the ongoing export is likely to restrict the time frame for possible interactions of the respective receptor/ligand complexes with nuclear chromatin. It also indicates that corresponding molecular gene activation might actually require several passages of shuttling (Fig. 1b & see below).

### Export of the AHR from the nucleus is strictly dependent on V647

**Figure 2:**
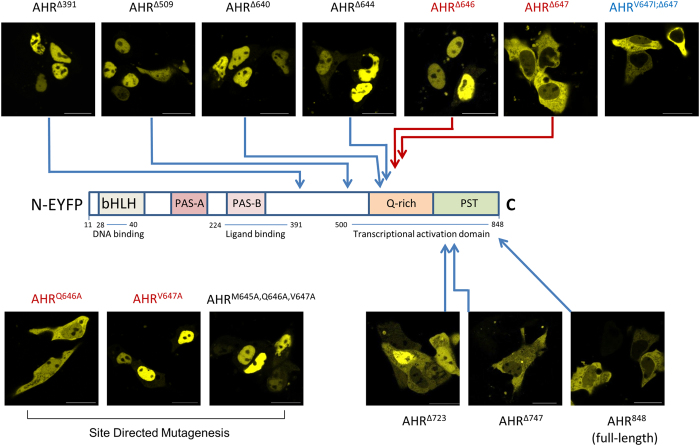
V647 determines the compartmentalisation of the AHR: expression of fluorescent AHR deletion mutants in HepG2 cells. Deletion mutants were derived from full-length human pEYFP-AHR-C1 (AHR^848^). Mutants are named according to truncations sites, as defined by the last included residue. These sites are marked on the drafted full-length protein. Representative images that reflect the typical compartmentalisation are shown (scalebar = 20 μm). Mutants truncated after amino acid 391 (AHR^∆391^), 509 (AHR^∆509^), 640 (AHR^∆640^) 644 (AHR^∆644^) and 646 (AHR^∆646^) show an exclusive nuclear staining, whereas the full-length protein (AHR^848^) is predominantly located in the cytosol. AHR^∆647^ is nearly exclusively detected in the cytoplasm. Inclusion of the Q-rich domain does increase nuclear association (AHR^∆723^). This is balanced by a motif localised between Pro 728 and Leu 744 within in the PST domain. AHR^∆647^ and full-length AHR^848^ show a similar predominantly cytoplasmic localisation. Replacing of residues M645, Q646 and V647 (AHR^M645A,Q646A,V647A^), or V647 only (AHR^V647A^) by alanines led to an exclusive nuclear staining, whereas mutant AHR^Q646A^ showed wild-type compartmentalisation (lower panel left). On the other side, replacement of V647 with isoleucine (AHR^V647I;∆647^) did not affect the cytoplasmic staining pattern (upper panel right).

**Figure 3:**
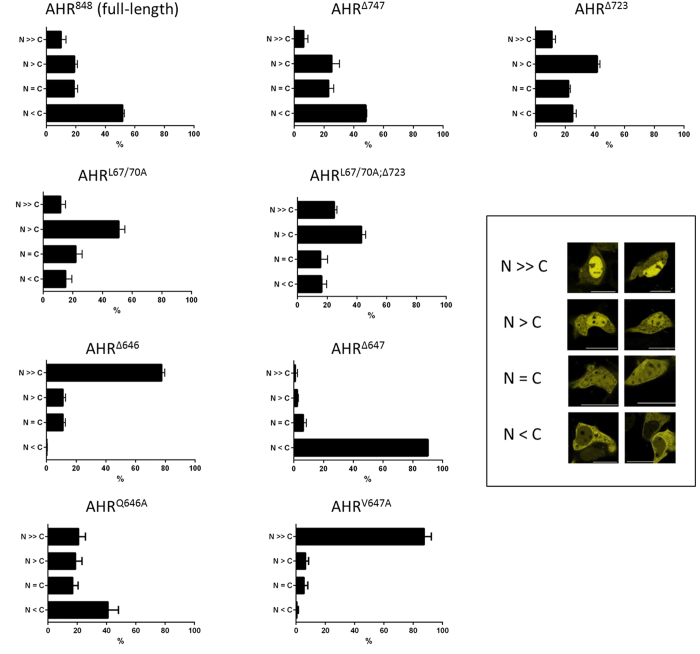
Compartmentalisation of EYFP-AHR mutants in transfected cells. A total of at least 300 positive cells that were found in randomly selected optical fields were analysed and classified after 24 h according to the defined staining patterns. Data represent the mean +/− S.D. out of three independent transfections. Insert: Staining patterns have been defined according to shown examples. N >> C exclusively nuclear; N > C predominantly nuclear; N = C equal distribution; N < C predominantly cytoplasmic.

Intracellular trafficking of the human AHR was further characterised using several deletion mutants. As previously observed for the murine protein by Pollenz and colleagues^26^, extensive C-terminal deletion of murine AHR led to accumulation in the nucleus. In our experiments, an exclusive nuclear detection was observed for human AHR^∆509^ which lacks 339 amino acids at the C-terminus, as well as for the shorter variant AHR^∆391^ (Fig. 2). This observation confirms constitutive basal import of the AHR as key mechanism in shuttling. In contrast, the N-terminal NES acts not autonomously, but apparently requires either some additional factors or structural elements of the C-terminal domain. We explored the latter option by analysing several fluorescent mutants, namely AHR^∆640^, AHR^∆644^, AHR^∆647^, AHR^∆650^, AHR^∆661^ and AHR^∆698^. The data show a striking difference between AHR^∆644^ and AHR^∆647^. The inclusion of these three residues was sufficient to shift nuclear staining to a nearly exclusive cytoplasmic state (Figs 2 and 3). Further site directed mutagenesis of residues 645 to 647 (Fig. 2, see AHR^M645A,Q646A,V647A^) led to an exclusive nuclear localisation of the full-length receptor. The respective locus seems therefore essential for any measurable export to happen. Further deletion mutagenesis confirmed V647 as critical residue (see AHR^∆646^, Figs 2 and 3).

We have applied additional site directed mutagenesis to confirm these findings (Fig. 2). While replacement of Q646 with alanine (AHR^Q646A^) did not alter the cytoplasmic staining pattern of full-length AHR, replacement of V647 with alanine (AHR^V647A^) did and led again to an exclusive nuclear staining. Interestingly, V647 is not conserved within in the AHR-sequence, but aligned to isoleucine in several species including mice. To test whether both amino acids are interchangeable, V647 was replaced with isoleucine in the AHR^∆647^ mutant, thus creating AHR^V647I;∆647^. In this experiment an exclusive cytoplasmic staining was confirmed similar to AHR^∆647^. Therefore our data suggest equivalent properties of valine and isoleucine at residue 647 of the human AHR.

### Activation-induced nuclear association of the AHR depends on the Q-rich domain

**Figure 4:**
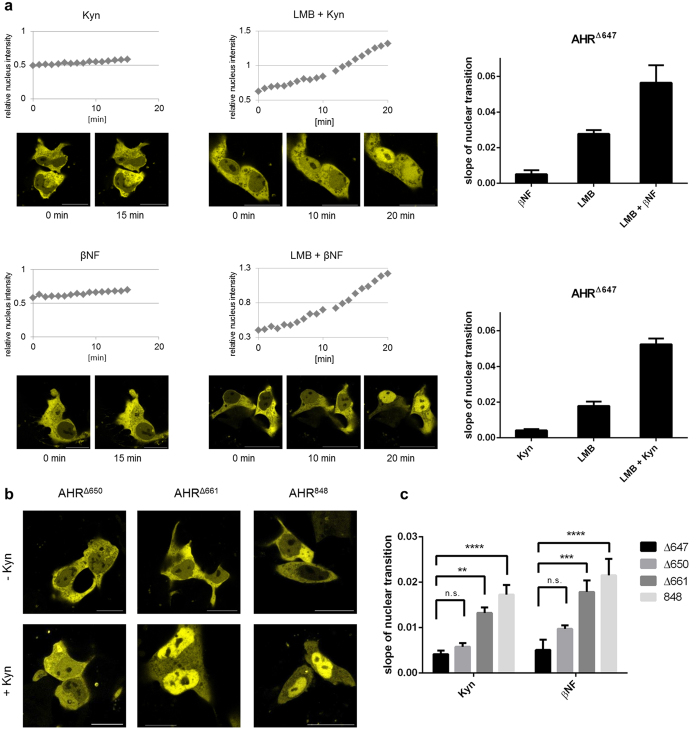
Ligand-induced nuclear association of the AHR depends on the Q-rich domain. (**a**) Translocation of EYFP-AHR^∆647^ into the nucleus in HepG2 cells after treatment with 100 μM Kyn or 10 μM βNF for 15 min (left). Translocation of AHR^∆647^ after exposure to 40 nM LMB for 10 min, followed by direct addition of 100 μM Kyn or 10 μM βNF for another 10 min (middle). Representative images of treated cells are shown for the indicated time points (scalebar = 20 μm). Nuclear transition was recorded and analysed as described in Fig. 1a. Slopes of the linear transition graphs have been separately determined for single and combined treatments (right). Each bar represents the mean +/− S.E.M. of 6 analysed cells. (**b**) Snapshots of cells imaged from transfected populations that were treated for 1 h with Kyn or left untreated. In response to ligand, AHR^∆661^ and full-length AHR^848^ showed a nearly exclusive nuclear staining pattern, while AHR^∆650^ remained predominantly cytoplasmic as in non-treated cells. Similar effects were observed after application of βNF. (**c**) Residues 648–661 are required for ligand-induced nuclear accumulation of the AHR. Cells expressing AHR^∆647^, AHR^∆650^, AHR^∆661^ or AHR^848^ (full-length) were treated with 100 μM Kyn or 10 μM βNF. Both, AHR^∆661^ and AHR^848^ showed significantly higher nuclear translocation rates than AHR^∆647^ (two-way ANOVA, **p < 0.01, ***p < 0.001, ****p < 0.0001). No such significant differences were observed between AHR^∆647^ and AHR^∆650^. Values depicted represent the mean +/− S.E.M of at least 5 cells.

The C-terminal deletion mutant AHR^∆647^ lacks essential parts of the Q-rich domain^28^. Notably this mutant showed an exclusive cytoplasmic staining that was markedly increased when compared with the wild-type receptor (Fig. 2). The deleted adjacent sequence is required for transcriptional activation of target genes *via* recruitment of co-activators and components of the basal transcription machinery^28^. Kinetic analysis of this mutant confirms its basal nuclear transition to be comparable to the full-length protein (Figs 1 and 4a). Similarly to the full-length receptor co-treatment with Kyn and the export inhibitor LMB accelerated the basal nuclear translocation, as did co-exposure to βNF and LMB. This was expected, since the deletion does neither affect the NLS, nor the ligand binding sites. Yet, contrastingly single substance exposure to Kyn or βNF failed to induce a stable or prolonged nuclear association of mutant AHR^∆647^ (Fig. 4a). These data indicate that the Q-rich domain is required for ligand-induced export inhibition, likely involving further protein interactions. Consequently, interactions of ligand-bound AHR-complexes *via* the N-terminal domain are not sufficient to stabilise a nuclear fraction beyond the transient levels of shuttling. These findings propose the Q-rich domain as crucial switch to exit shuttling. Further analysis specified residues 648–661 as sufficient to maintain a stable nuclear fraction during activation (Fig. 4b,c). For these mutants, we have analysed the kinetics of nuclear transfer in the presence of agonists in detail. Our data demonstrate that the section between residues 648–661 is required and sufficient to facilitate nuclear accumulation of the AHR in response to ligands (Fig. 4c). Still, nuclear transition of the full-length receptor was slightly enhanced, pointing to the possibility that additional sites of the transactivation domain stabilise the nuclear fraction during ligand-induced activation.

### The C-terminal PST domain affects the nucleocytoplasmic distribution of human full-length AHR

Microscopic analyses of AHR^∆723^ revealed a strongly enhanced nuclear staining in relation to AHR^∆647^ (Figs 2 and 3). Importantly, this staining was also distinguishable from the predominantly cytoplasmic pattern of the full-length AHR with its additional 125 amino acids. This finding raised the question on how the predominant cytoplasmic compartmentalisation is maintained. We analysed the C-terminal domain for relevant motifs, including potential export signals using LocNES predictor software^29^. The analysis yielded two high scoring hits that comprise two overlapping sequence motifs between Pro 728 and Leu 744 (Supplementary Fig. S3, PYPTTSSLEDFVTCLQL). Notably these motifs were not found in the partially homologous murine sequence, in which an alternate putative NES is predicted further upstream between Val 598 and Leu 612 by LocNES instead. Intriguingly, elongation of the human AHR protein to residue 747 (AHR^∆747^) restores the cytoplasmic compartmentalisation that is also seen with the wild-type receptor (AHR^848^) (Fig. 2). Kinetic analysis of basal nucleocytoplasmic shuttling and ligand-induced nuclear translocation further confirmed the similarity to full-length AHR (Supplementary Fig. S2a). In contrast, the predominant nuclear compartmentalisation of AHR^∆723^ was further increased by concomitant inactivation of the N-terminal NES (AHR^L67/70A;∆723^). Deletion of both motifs doubled the proportion of cells that showed a nearly exclusive nuclear staining (Fig. 3). However, when this motif (i.e. Pro 728 – Leu 744) was fused to the C-terminal domain of EYFP, it did not shift the fusion protein towards an enhanced cytoplasmic distribution and no nuclear accumulation was observed after treatment with LMB (data not shown). Accordingly, a possible function as autonomous CRM1-dependent export signal could not be substantiated in this study. The observed effects on the cytoplasmic fraction therefore might rather depend on upstream elements, especially the N-terminal NES and the Q-rich domain.

## Discussion

Functions of the AHR are closely related to intracellular trafficking both in the context of shuttling and ligand-induced nuclear transfer. According to the classical nuclear transport model, the AHR is complexed with Hsp90 and co-chaperones. Ligand binding had previously been proposed to stimulate release of Hsp90 from the N-terminal bHLH domain thus exposing the NLS and inducing transport of the AHR into the nucleus^30^,^19^. In addition, Richter and co-workers (2001) demonstrated that nuclear import can occur in the absence of xenobiotic ligands, but less efficiently^31^. Our data are in agreement with these previous observations and confirm a substantial acceleration of basal import by Kyn and βNF. Import of the AHR into the nucleus might also depend on endogenous ligands that could be formed in cultured cells as well. It seems conceivable that this process operates continuously, although less efficient in the absence of interacting ligands. According to the model proposed by Lees and Whitelaw (1999) interactions between Hsp90 and the bHLH are comparatively weak^30^. Therefore it might be well possible that the NLS is transiently exposed in a steady state proportion of receptor molecules that enter the nucleus.

**Figure 5:**
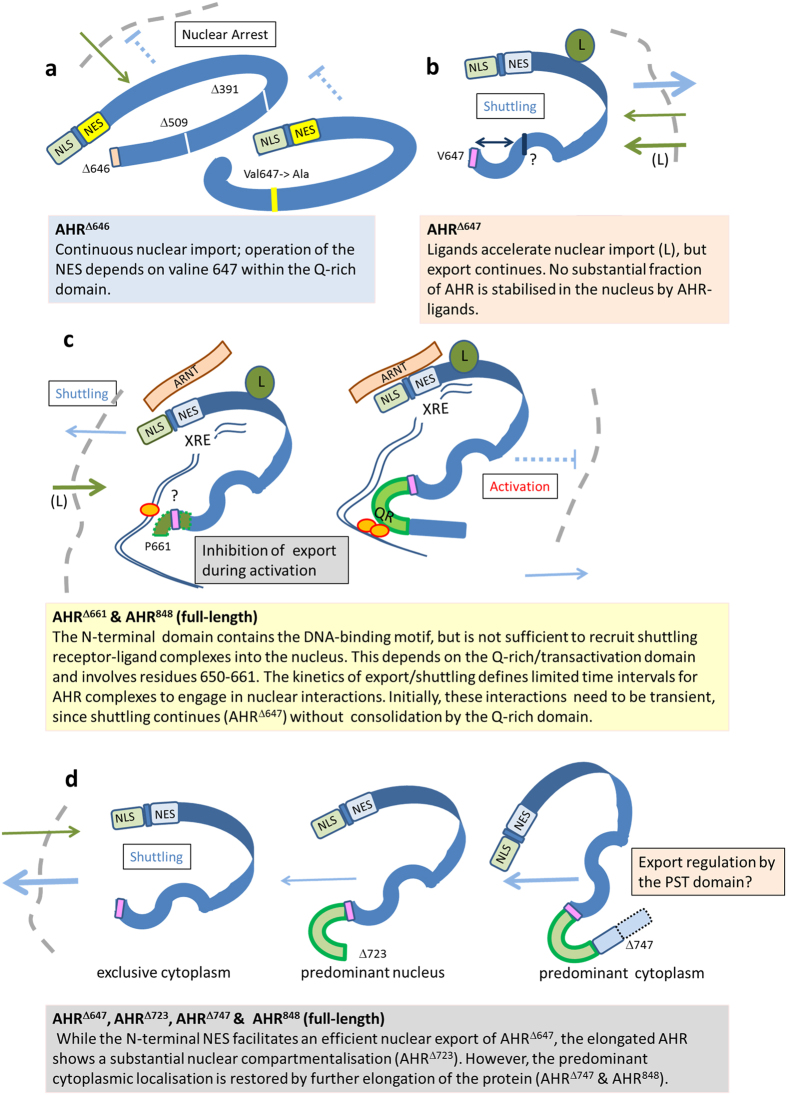
Nuclear export and intracellular trafficking of the human AHR are regulated by defined motifs. (**a**) The nuclear localisation signal (NLS) within the N-terminal domain triggers continuous basal import (green arrows) into the nucleus (shuttling). Contrary to this autonomous import mechanism, function of the adjacent nuclear export signal (NES) depends on C-terminal motifs, especially the mandatory residue V647. (**b**) Ligands (marked with L) accelerate import, while continued export (blue arrow) counteracts nuclear sequestration of the AHR, thus maintaining a predominant cytoplasmic fraction that is receptive for interactions with ligands. Notably, mutants that lack parts of the C-terminal domain (AHR^Δ647^ and AHR^Δ650^) do not efficiently accumulate in the nucleus, although nuclear transfer is accelerated by ligands. (**c**) Export of the AHR continues in the presence of ligands. Activation of the AHR might involve several passages of receptor molecules that need to engage in further associations with nuclear components during limited time intervals. Stable associations of the AHR with the nucleus likely require a defined section of the Q-rich domain (green, Pro 661 is indicated). However, it is as yet completely unknown how this motif stabilises nuclear compartmentalisation or whether it promotes interactions of the transactivation domain with transcription factors. (**d**) The N-terminal NES and the V647 motif facilitate an efficient nuclear export of AHR^Δ647^, leading to a nearly exclusive cytoplasmic pattern. On the other side, the full-length AHR contains an additional motif within the PST domain to maintain a predominantly cytoplasmic compartmentalisation.

The interplay between parallel import and export pathways provides a new perspective on the activation of the AHR shifting the focus from a sequential model to kinetic shuttling as a means of highly complex and fine-tuned receptor regulation. Firstly, the exclusive nuclear detection of deletion mutants supports the concept of a constitutively active NLS, which is also in agreement with rapid nuclear accumulation after export inhibition in LMB-treated cells^19^,^20^,^31^. Continual activation-independent export is therefore required to maintain a receptive cytosolic fraction of AHR complexes in waiting to respond to endogenous or xenobiotic (exogenous) ligands. Secondly, during activation, shuttling might assert an element of kinetic control not only balancing the molecule pools of AHR between different intracellular compartments (nucleus *vs.* cytoplasm), but also by crucially limiting the time frame for nuclear interactions of both the N-terminal and Q-rich domains in a ligand-dependent manner. We have summarised our conclusions in accordance to the analysed AHR mutants in Fig. 5. Importantly, the N-terminal NLS acts autonomously. Although it might be masked by interacting factors, no particular conformation or regulation by other AHR domains is apparently required for its functions. According to our data, this is a marked difference to the NES that is also localised in the N-terminal domain. The capacity of the N-terminal NES to shift AHR complexes out of the nucleus depends on a C-terminal section within the Q-rich domain that comprises V647 as mandatory residue. Possibly, this residue stabilises a conformation required for nuclear export, but apparently not for import. Further, the same region of the Q-rich domain is essential to consolidate a stable nuclear association during receptor activation. Again, this does not necessarily involve interactions with transcription factors or other proteins, but might be alternatively related to structural properties of the full-length protein.

Taken together, we have defined a section of the Q-rich domain that regulates export and trafficking of the AHR in the context of both nucleocytoplasmic shuttling and receptor activation. In addition, export regulation that involves the essential N-terminal NES is also affected by a further C-terminal motif within the PST domain.

## Methods

### Reagents and plasmids

Dimethyl sulfoxide (DMSO), β-naphthoflavone (βNF), leptomycin B (LMB), and kynurenine (Kyn) were obtained from Sigma-Aldrich (Sigma-Aldrich Chemie GmbH, Munich, Germany). Full-length or truncated cDNA of human AHR were subcloned into pEYFP-C1 (Clontech), using the Bgl*II* and Kpn*I* sites. A unique forward primer 5′-catgacagatctgccagtcgcaagcggcggaag-3′ was used. Constructs encode the processed human AHR starting at Ala 11.

As reverse primers the following oligonucleotides were used. Full-length AHR^848^ (11–848): 5′-catgacggtaccttacaggaatccactggatgtcaaatc-3′; Truncated AHR variants:

AHR^∆391^: 5′-acacaggtaccttatccttcctcatctgttagtgg-3′;

AHR^∆509^: 5′-acacaggtaccttacaggatagtatcatttcccatc-3′;

AHR^∆640^: 5′-acacaggtaccttactgacacagctgttgctgtgg-3′;

AHR^∆644^: 5′-acacaggtaccagatctttagtgcttcatcttctgacacagctg-3′;

AHR^∆646^: 5′- acacaggtaccgaattcttattgcatgtgcttcatcttctgacac-3′;

AHR^∆647^: 5′-acacaggtaccaagcttaaacttgcatgtgcttcatcttctgacac-3′;

AHR^∆650^: 5-acaca ggtaccttacatgccattaacttgcatgtgc-3

AHR^∆661^: 5-acacaggtaccagatcttaaggcacgaattggttagagttcc-3

AHR^∆698^: 5′-acacaggtaccttactgtgtataaggcatagaatcc-3′

AHR^∆723^: 5′-acacaagcttggtaccttaactccccatagggtagtccagctc-3′;

AHR^∆747^: 5′-acacaggtaccttagttttcaggaagttgtaaacaagtg-3′;

AHR^∆808^: 5′-acacaggtaccttaatttaaaactccattctgaaacttg-3′.

AHR^V647I;∆647^: 5′-acacaggtaccaagcttaaatttgcatgtgcttcatcttctgacac-3′;

pEYFPAHR^∆NES^ variants that carry mutations at certain amino acids (AHR^L67/70A^, AHR^M645A,Q646A,V647A^, AHR^Q646A^, AHR^V647A^) were generated and sequenced by MGW Eurofins (Ebersberg, Germany).

### Tissue culture and treatments

The human hepatoma cell line HepG2 was cultured in RPMI. Human embryonic kidney 293 (HEK293) cells were cultured in DMEM. Both cell lines were maintained in 5% CO_2_ at 37 °C in culture medium containing 10% fetal calf serum (v/v), L-glutamine (2 mM), and penicillin/streptomycin (100 U/ml). All media components were purchased from Pan-Biotech (Aidenbach, Germany). For stimulation of cells, the media were replaced with fresh media or HBSS (Gibco - Thermo Fisher, Waltham, MA, USA) containing 10 μM βNF, 100 μM Kyn or 40 nM LMB dissolved in DMSO/ethanol and further incubated for the indicated time period. Control cells were treated with solvent vehicle (0.1% DMSO) only.

### Transient transfection

Depending on the further application, HepG2 cells and HEK293 cells were seeded either on 6-well plates (Techno Plastic Products AG, Trasadingen, Switzerland), glass-bottom dishes (*In Vitro* Scientific, Sunyvale, CA, USA), or on cover glasses (ThermoFisher Scientific, Loughborough, UK). At next day they were transfected with Lipofectamine 2000 (Invitrogen, Carlsbad, CA, USA) and an appropriate DNA concentration according to the manufacturer’s instructions. For HEK293 cells, the surfaces were coated with poly-L-lysine (Biochrom AG, Berlin, Germany).

### RNA analysis

RNA was isolated using RNeasy Mini kit in connection with the QIAshredder (QIAGEN GmbH, Hilden, Germany). The purity and the concentration of each RNA sample were determined using a NanoDrop1000 device (Peqlab Biotechnologie GmbH, Erlangen, Germany). The isolated RNA was used for cDNA synthesis with the High Capacity RNA-to-cDNA kit (Applied Biosystems, Foster City, CA, USA). Real-time PCR was carried out using an SYBR green assay (5Prime, Hamburg, Germany). Hypoxanthine guanine phosphoribosyl transferase (HPRT) was used as reference gene.

### Western-blot analysis

Cells were lysed on ice in RIPA buffer containing 50 mM Tris/HCl, pH 7.4, 150 mM NaCl, 1 mM EDTA, 1% Igepal^®^ and 0.25% sodium deoxycholate, with protease inhibitor cocktail (Calbiochem, San Diego, CA, USA). Equal amounts of proteins were applied to SDS-PAGE, transferred onto nitrocellulose membranes and immunoblotted according to the manufacturer’s instructions. The primary antibody against AHR was used at 1:200 (sc-5579; Santa Cruz Biotechnology, Santa Cruz, CA, USA). Primary antibody probed blots were visualised with appropriate horseradish peroxidase-coupled secondary antibodies (Santa Cruz Biotechnology) using enhanced chemiluminescence (34078; Thermo Scientific, Waltham, MA, USA) for detection.

### Immunofluorescence staining

For immunofluorescence staining the Inside Stain Kit (Miltenyi Biotech Ltd., Bisley, Surrey, UK; Miltenyi Biotech Inc., Auburn, CA, USA) was used with minor modifications. After the fixation step, the cover glasses were incubated with the anti-AHR primary antibody (1:100, Santa Cruz Biotechnology) overnight at 4 °C. The slides were then washed and incubated with the secondary antibody for 30 min at room temperature (1:500, anti-rabbit FITC, Bethyl Laboratories, Inc., Montgomery, TX, USA). The slides were mounted in the VECTASHIELD HardSet Mounting Medium with DAPI (VECTOR LABORATORIES, INC., Burlingame, CA, USA), and then analysed using a confocal microscope (see below).

### On-line confocal microscopy

For live-cell fluorescence imaging microscopy, HepG2 cells were seeded on glass-bottom dishes and transfected with plasmid DNA. Twenty four hours post transfection, fresh medium was applied and cells were monitored by confocal microscope. For on-line investigations, representative cells or cell groups were selected and maintained in buffered medium at 37 °C. Concurrently with treatments, live-cell imaging was started at a rate of one picture per minute. Typically, single treatment experiments were finalised after 15 min and combined treatment experiments after 20 min. Microscopic analyses and image acquisitions were done on an LSM 700 confocal microscope (Carl Zeiss Jena GmbH, Jena, Germany), using ZEN 2012 blue edition and ZEN 2011 black edition software (Carl Zeiss Jena GmbH). Data were analysed and graphed using Microsoft Excel and Prism Software (Graph Pad, La Jolla, CA, USA). Statistical analysis was done using two-way ANOVA and either Dunnett’s or Sidak’s multiple comparisons test. α = 0.05; **p < 0.01, ***p < 0.001, ****p < 0.0001.

### Cloning and expression of AHR

The human AHR DNA sequence was cloned into the pTFCold vector system (Clonetech, Takara) using a synthesised and codon-optimised construct. Clone identity was confirmed by sequencing and the respective plasmids where subsequently used for protein expression in the Rosetta 2 (DE3) system (Novagen) using 2xYT-medium and standard conditions with 300 μM isopropyl β-D-1-thiogalactopyranoside (IPTG), as recommended by the manufacturer. Following overnight cold-expression cells were harvested, washed (20 mM PIPES, 300 mM NaCl, pH 7.8) and subjected to lysis by sonication. At least 70% of expressed AHR were recovered in the soluble fraction and purified using Talon superflow agarose (GE Healthcare, Freiburg, Germany) according to manufacturer’s instructions. After elution the protein concentration was estimated by spectrophotometric analysis and the purification efficiency was checked using SDS-PAGE (refer to Supplementary Fig. S4 for an exemplary gel).

### Thermal shift assay

Thermal stability of AHR without and with ligands was assessed fluorometrically as described^32^. Assays were performed in a 96-well format using SYPRO orange in conjunction with the melting curve feature of a real-time PCR cycler. Following background subtraction the melting point was calculated based on the maxima of the melting curves using the equation of Biggar and co-workers^32^.

### Matrix-assisted laser desorption/ionisation Time-of-Flight (MALDI-ToF)

We used MALDI-ToF to verify the identity of the recombinant human AHR protein. Following electrophoretic separation gel slices containing recombinantly expressed and purified AHR (~150 kDa) were cut out, destained, trypsin digested and subjected to fragment-pattern analysis using the following protocol. Destained gel slices were first reduced for 15 min at 60 °C using 100 mM ammonium carbonate with 45 mM dithiothreitol (DTT), and then alkylated for 15 min using 100 mM iodoacetamide. Following alkylation the gel slice was washed for three times and equilibrated for another 30 min in equilibration buffer containing 50 mM ammonium bicarbonate and 5% acetonitrile before commencing with overnight 4 ng/μL trypsin digestion at 37 °C. Following digestion the samples were extracted with 60% acetonitrile/0.1% trifluoroacetic acid (TFA) and 100% acetonitrile. Subsequently, samples were purified and desalted using C18 ZipTips (Merck Millipore, Darmstadt, Germany) with TFA supplemented (0.1%, v/v) washing solution. Eluted samples were spotted with α-cyano-4-hydroxycinnamic acid matrix on AnchorChip targets (Bruker, Rheinstetten, Germany) and analysed using an UltrafleXtreme MALDI-ToF/ToF (Bruker, Rheinstetten, Germany). Data evaluation was performed with ProteinScape (MASCOT/Swissprot database). The following search parameters were used: 1 missed cleavage, carbamidomethyl (Cys) as fixed modification and oxidation (Met) as variable modifications. Taxonomy was set to *Homo sapiens*. MS tolerance and MS/MS tolerance were set to 50 ppm and to 0.7 Da, respectively.

## Additional Information

**How to cite this article**: Tkachenko, A. *et al*. The Q-rich/PST domain of the AHR regulates both ligand-induced nuclear transport and nucleocytoplasmic shuttling. *Sci. Rep.* **6**, 32009; doi: 10.1038/srep32009 (2016).

## Supplementary Material

Supplementary Information
