# Supplementary material for: The Q-rich/PST domain of the AHR regulates both ligand-induced nuclear transport and nucleocytoplasmic shuttling
Source: Sci Rep. 2016 Aug 18;6:32009. doi: 10.1038/srep32009 (PMC4989234; doi:10.1038/srep32009)
Supplement: Supplementary Information [file srep32009-s1.pdf]

Supplementary information to:

The Q-rich/PST domain of the AHR regulates both ligand-induced nuclear transport and nucleocytoplasmic shuttling

Anna Tkachenko[†], Frank Henkler[†], Joep Brinkmann, Juliane Sowada, Doris Genkinger, Christian Kern, Tewes Tralau, and Andreas Luch*

Affiliation: German Federal Institute for Risk Assessment (BfR), Department of Chemical and Product Safety, Max-Dohrn-Strasse 8-10, 10589 Berlin, Germany.

*Corresponding author. Email: Andreas.Luch@bfr.bund.de; phone: ++49-30-18412-4538.

[†]These authors contributed equally to this work.

Fig. S1

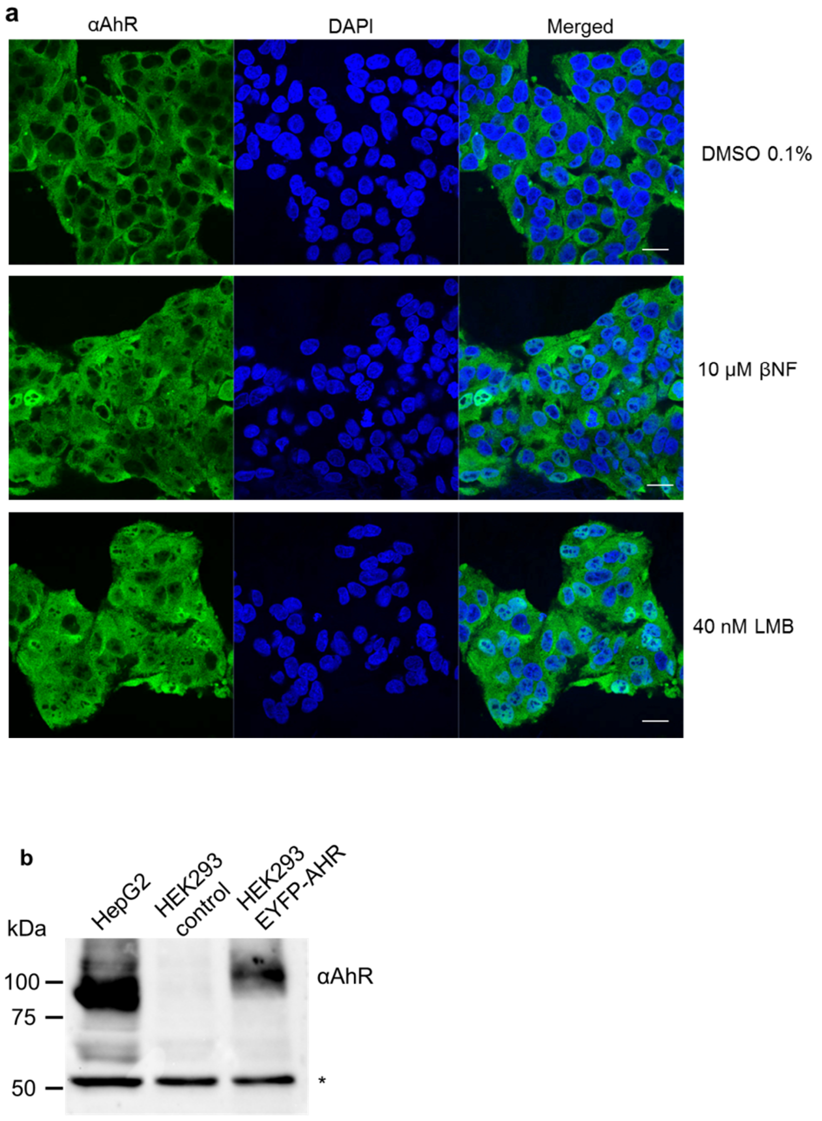

Figure S1. (**a**) Immunofluorescence staining of endogenous wild-type AHR in HepG2 cells after 1 h treatment with 10 µM βNF and 40 nM LMB (αAHR 1:100). Scalebar = 20 µm. βNF and LMB induce a similar nuclear translocation of the endogenous AHR. (**b**) Western-blot analysis of transfected human EYFP-AHR fusion protein in HEK293 cells, compared with endogenous AHR in HepG2 cells. *Cross-reaction bands indicate equal loading.

Fig. S2

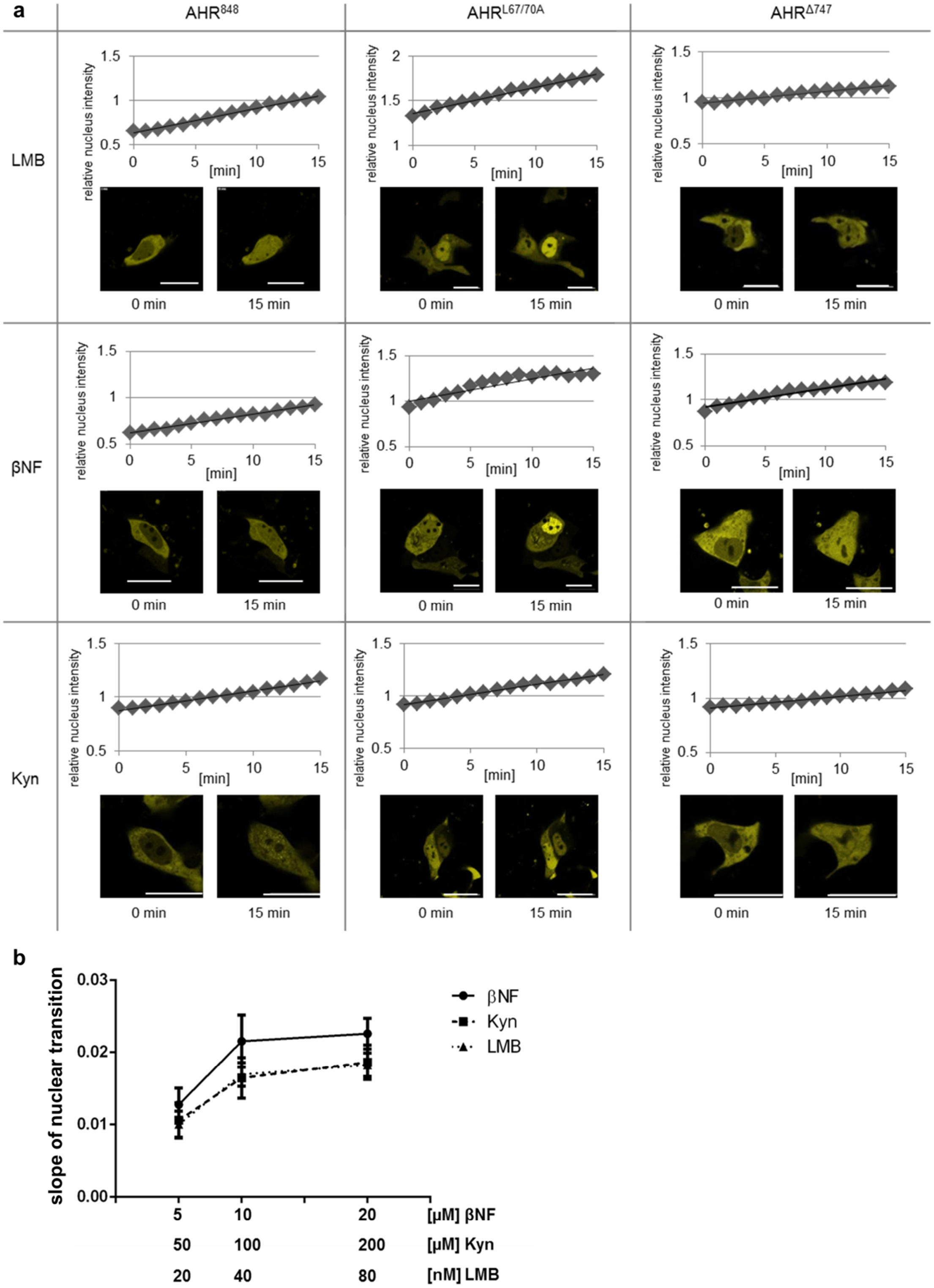

Figure S2. (**a**) Localisation and shuttling of full-length AHR (AHR^{848}), $AHR^{L67/70A}$ and $AHR^{\Delta747}$. Kinetics of protein translocation into the nucleus are shown during 15 min after treatment with 40 nM LMB, 10 µM βNF, or 100 µM Kyn. (**b**) Dose-dependent increases of the relative nucleus fluorescence intensity induced by βNF, LMB and Kyn. Cells treated with 10 µM βNF, 40 nM LMB and 100 µM Kyn show a similar nuclear fluorescence intensity increase, which significantly differs from solvent (DMSO) control cells (two-way ANOVA, p<0.0001=****). Values depicted represent the mean +/- S.E.M of 6 cells.

Fig. S3

Score human LocNES 510 – 748 (Gln 640 is labelled red):

KHEQIDQPQDVNSFAGGHPGLFQDSKNSDLYSIMKNLGIDFEDIRHMQNEKFFRNDFSGEVDFRDIDLTDEILTYV
QDSLSKSPFIPSDYQQQQSLALNSSCMVQEHLHLEQQQQHHQKQVVVEPQQQLCQKMKHMQVNGMFENWN
SNQFVPFNCPQQDPQQYNVFTDLHGISQEFPYKSEMDSMPYTQNFISCNQPVLPQHSKCTELDYPMGSFEPSPYP
TTSSLEDFVTCLQLPENQ

Protein Name	Position	Sequence	Score
>LocNES69844683_0	25-39	SKNSDLYSIMKNLGI	0.122
>LocNES69844683_0	30-44	LYSIMKNLGIDFEDI	0.152
>LocNES69844683_0	49-63	NEKFFRNDFSGEVDF	0.014
>LocNES69844683_0	54-68	RNDFSGEVDFRDIDL	0.026
>LocNES69844683_0	62-76	DFRDIDLTDEILTYV	0.030
>LocNES69844683_0	124-138	EPQQQLCQKMKHMQV	0.025
>LocNES69844683_0	219-233	PYPTTSSLEDFVTCL	0.338
>LocNES69844683_0	221-235	PTTSSLEDFVTCLQL	0.350

Figure S3. Analysis of the C-terminal domain of the human AHR protein, using the LocNES computational tool[29].

Fig. S4

a

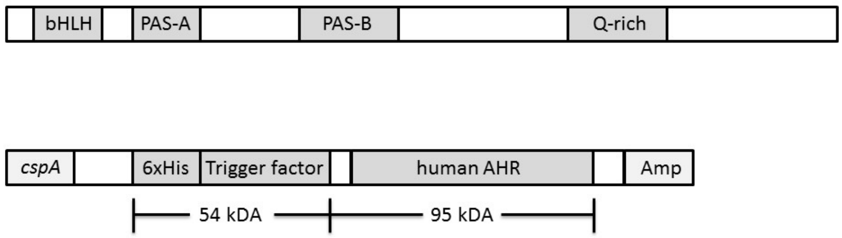

b

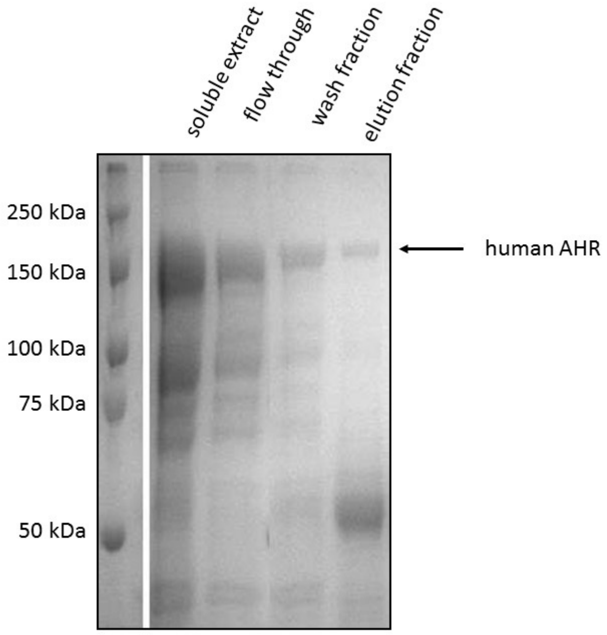

Figure S4. AHR expression construct for protein purification. (**a**) Domain structure of the human AHR. The bHLH, PAS and Q-rich domains are marked. A sketch of the generated AHR expression construct is given below. (**b**) Exemplary SDS-PAGE of AHR purification. The eluted fraction contains AHR protein and its degradation products as confirmed by MALDI-ToF (see section Methods).
